# Age Cohort Differences and Depressive Symptoms Among Community-Dwelling Older Americans

**DOI:** 10.1093/geroni/igaa057.199

**Published:** 2020-12-16

**Authors:** Ethan Siu Leung Cheung, Ada Mui

**Affiliations:** 1 Columbia University, Bronx, New York, United States; 2 Columbia University, New York, New York, United States

## Abstract

This study uses Wave 3 National Social Life, Health and Aging Project to examine the correlation between age cohorts [60s (n=1204); 70s (n=1176); 80 and older (n= 724)], cognitive status, and depression symptoms. In the total sample, 53.90% were females, 76.15% Whites, 15.29% Blacks, and 8.56% Asians. Compared to the 60s and 70s cohorts, 80+ cohort was cognitively more impaired [Mean (SD) of MoCA Short Form were 10.7(2.9), 10.0(3.2), and 8.1(3.6)]. There were no age cohorts’ differences in depressive symptoms experienced (Mean of CESD Short Form = 21.03; SD = 4.06). In order to identify predictors of depression, multiple hierarchical regressions were performed. The 60s sample was the reference group to compare with 70s and 80s cohorts. Results showed that age cohort variables had a significant independent effect as well as a joint effect with cognitive status in explaining depression scores. For each age cohort group, parallel regression analyses were conducted and all models were significant. Findings suggest that ADL impairment was the only common predictor for depressive symptoms for the three cohort groups, and the association was the strongest for the 60s cohort (b = .31). Other unique predictors for 60s cohort were lower-income, more IADLs impairment, higher stress and cognitive impairment. For the 70s cohort, unique predictors of depressive symptoms were female gender, unmarried, and less socialization. For the 80 and above group, correlates of depression are female, White, and high stress level. Findings highlight the necessity of age-sensitive programs on depression support for community-dwelling older Americans.

